# DNA methylation‐based profiling of bone and soft tissue tumours: a validation study of the ‘DKFZ Sarcoma Classifier’

**DOI:** 10.1002/cjp2.215

**Published:** 2021-05-05

**Authors:** Iben Lyskjær, Solange De Noon, Roberto Tirabosco, Ana Maia Rocha, Daniel Lindsay, Fernanda Amary, Hongtao Ye, Daniel Schrimpf, Damian Stichel, Martin Sill, Christian Koelsche, Nischalan Pillay, Andreas Von Deimling, Stephan Beck, Adrienne M Flanagan

**Affiliations:** ^1^ Research Department of Pathology University College London, UCL Cancer Institute London UK; ^2^ Medical Genomics Research Group University College London, UCL Cancer Institute London UK; ^3^ Department of Histopathology Royal National Orthopaedic Hospital Stanmore UK; ^4^ Department of Neuropathology University of Heidelberg Heidelberg Germany; ^5^ Clinical Cooperation Unit Neuropathology, German Cancer Consortium (DKTK) German Cancer Research Center (DKFZ) Heidelberg Germany; ^6^ Hopp‐Children's Cancer Center (KiTZ) Heidelberg Germany; ^7^ Division of Pediatric Neurooncology, German Cancer Consortium (DKTK) German Cancer Research Center (DKFZ) Heidelberg Germany; ^8^ Department of General Pathology University of Heidelberg Heidelberg Germany

**Keywords:** bone, classifier, methylation profiling, soft tissue, sarcoma

## Abstract

Diagnosing bone and soft tissue neoplasms remains challenging because of the large number of subtypes, many of which lack diagnostic biomarkers. DNA methylation profiles have proven to be a reliable basis for the classification of brain tumours and, following this success, a DNA methylation‐based sarcoma classification tool from the Deutsches Krebsforschungszentrum (DKFZ) in Heidelberg has been developed. In this study, we assessed the performance of their classifier on DNA methylation profiles of an independent data set of 986 bone and soft tissue tumours and controls. We found that the ‘DKFZ Sarcoma Classifier’ was able to produce a diagnostic prediction for 55% of the 986 samples, with 83% of these predictions concordant with the histological diagnosis. On limiting the validation to the 820 cases with histological diagnoses for which the DKFZ Classifier was trained, 61% of cases received a prediction, and the histological diagnosis was concordant with the predicted methylation class in 88% of these cases, findings comparable to those reported in the DKFZ Classifier paper. The classifier performed best when diagnosing mesenchymal chondrosarcomas (CHSs, 88% sensitivity), chordomas (85% sensitivity), and fibrous dysplasia (83% sensitivity). Amongst the subtypes least often classified correctly were clear cell CHSs (14% sensitivity), malignant peripheral nerve sheath tumours (27% sensitivity), and pleomorphic liposarcomas (29% sensitivity). The classifier predictions resulted in revision of the histological diagnosis in six of our cases. We observed that, although a higher tumour purity resulted in a greater likelihood of a prediction being made, it did not correlate with classifier accuracy. Our results show that the DKFZ Classifier represents a powerful research tool for exploring the pathogenesis of sarcoma; with refinement, it has the potential to be a valuable diagnostic tool.

## Introduction

Bone and soft tissue tumours are rare, with sarcomas, comprising approximately 100 different subtypes, representing no more than 2% of all cancers. Histological assessment has been the bedrock of tumour classification for the last 200 years [1], but advances in next‐generation sequencing technology, in combination with a greater understanding of the mechanism of disease at a molecular level, have led to a significant refinement of cancer classification. In addition to improving diagnostic accuracy, categories based on molecular findings can offer additional information enabling clinicians to provide more informed prognoses and discuss evidence‐based treatment options with their patients. Despite significant advances in the classification of bone and soft tissue tumours, there remains a large group of sarcomas with no defining molecular hallmarks, the diagnosis of which remains based purely on morphological interpretation. Indeed, it is still not uncommon to be unable to provide a specific diagnosis, and this contributes to the lack of improved outcomes for patients with sarcoma over the past 40 years [2].

DNA methylation profiles are now regularly employed as part of the toolkit for classifying brain tumours [3, 4], the success of which prompted a similar approach to classify sarcomas resulting in the recently published ‘Deutsches Krebsforschungszentrum (DKFZ) Sarcoma Classifier’ [5]. This classifier was built using methylation profiles of 1,077 reference samples representing 54 bone and soft tissue tumour subtypes as well as common mimics of sarcoma and normal control tissues. Based on their initial validation cohort of 428 samples, Koelsche *et al* reported that 75% of cases obtained a successful diagnostic prediction based on their methylation profiles. The majority of predicted methylation classes (91%) were concordant with the original histological diagnosis, and 9% of predictions resulting in a revised histological diagnosis in favour of the predicted methylation class after histological review and confirmation by relevant molecular tests [5].

Our work and that of others on DNA methylation profiling of sarcoma has demonstrated key insights into specific tumour types [6, 7, 8, 9, 10, 11], and has shown that DNA methylation can add value to whole‐genome and RNA sequencing data. To enhance the benefit of the genomes delivered from 1,200 patients with sarcoma as part of the UK's 100,000 Genomes Project [12], we have undertaken methylation profiling of a significant proportion of these patients' samples, with the aim of providing greater insight into the pathogenesis of sarcoma and its mimics. Improved classification of sarcoma should not only reap benefits in the clinical setting, but also provide new angles from a research perspective. To this end, we used our methylation data set generated from 986 samples to validate the performance of the DKFZ Sarcoma Classifier v12, hereafter referred to as the DKFZ Classifier, the first of its kind for these rare diseases.

## Methods

Ethical approval was obtained from the Cambridgeshire 2 Research Ethics Service (reference 09/H0308/165). All samples were collected through the UCL Biobank for Health and Disease at the Royal National Orthopaedic Hospital (RNOH, Stanmore, UK), which is covered by Human Tissue Authority licence 12055: project EC17.14. Samples included in this study were diagnosed at the RNOH between 2003 and 2019, and were assigned using the World Health Organization (WHO) classification criteria available at the time of diagnosis. Only samples with a tumour content of at least 40% were subjected to DNA methylation analysis (Infinium HumanMethylation450 or EPIC array; Illumina, San Diego, CA, USA).

### DNA methylation data set

DNA methylation profiling data were available from 986 samples; many of the samples were included in other studies over the last 5 years including the 100,000 Genomes Project [6, 7, 8, 9, 13]. For details of generation of these data, see Supplementary materials and methods. This data set comprised 929 bone and soft tissue tumour samples (see supplementary material, Table S1), six non‐mesenchymal tumour samples, as well as normal controls (blood and non‐neoplastic bone, nerve, and muscle, *n* = 51). Raw DNA methylation data files have been deposited in the ArrayExpress database at EMBL‐EBI (www.ebi.ac.uk/arrayexpress) under accession number E‐MTAB‐9875.

Raw iDAT files for all 986 samples were uploaded to the DKFZ Sarcoma Classifier (version 12) (www.molecularsarcomapathology.org). All classifier results consisted of a suggested methylation class with an accompanying calibrated score. The calibrated score is a probability of the confidence for the given methylation class assignment. As defined by Koelsche *et al*, the classifier was only deemed to have made a successful prediction if the sample obtained a calibrated score of 0.9 or higher [5].

**Figure 1:**
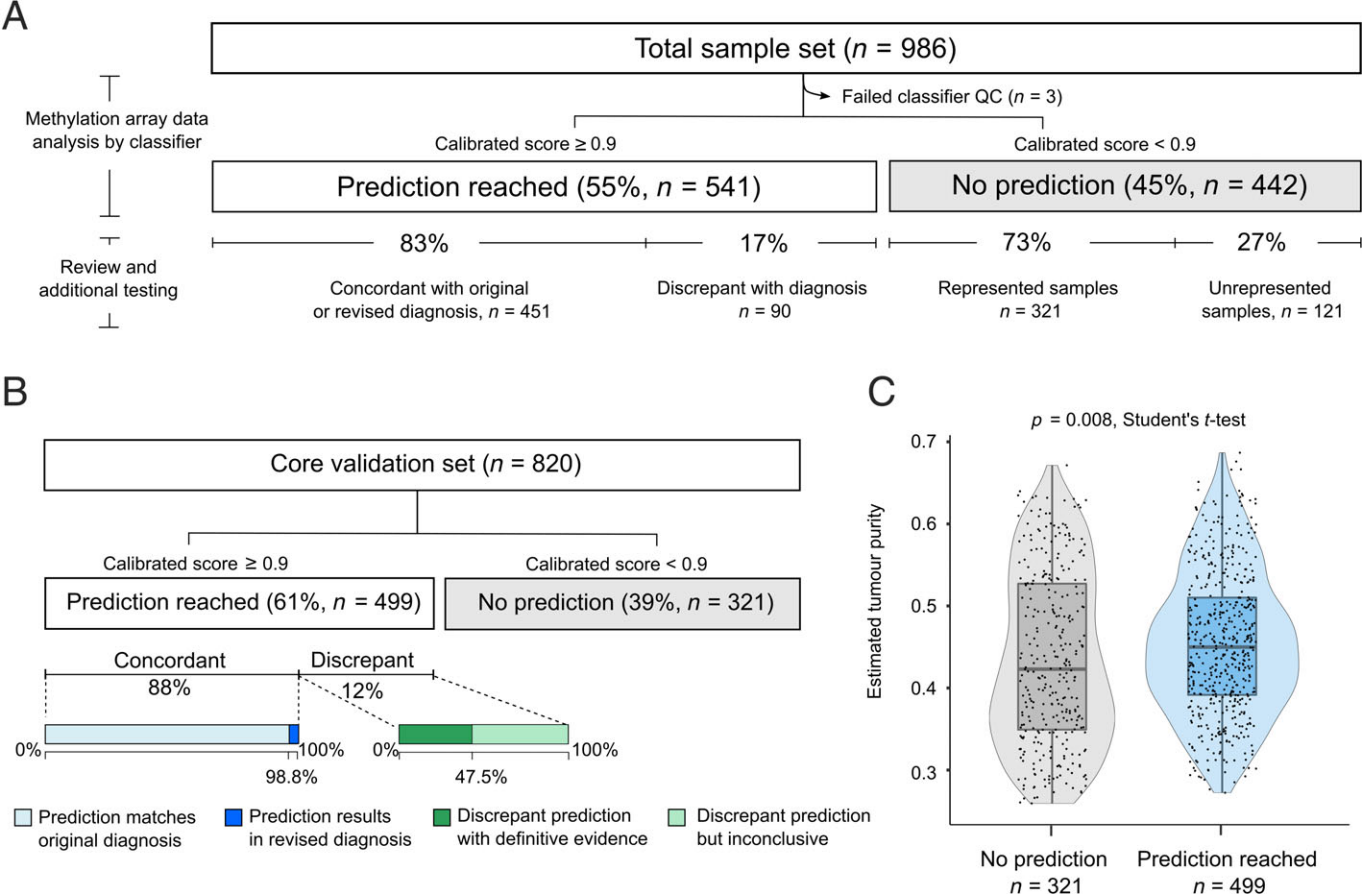
Overview of performance of the ‘DKFZ Classifier’ on the RNOH validation data set. (A) Overview of all cases in the study. (B) Overview of cases from the core validation cohort. (A and B) Prediction: classifier result with a calibrated score ≥0.9. The calibrated score is the probability for the given methylation class assignment. QC, quality control *Concordant*: samples predicted by the classifier to the methylation class corresponding with the original or revised diagnosis. *Discrepant*: where the predicted methylation class did not match the original histological diagnosis, and following review there was either sufficient evidence to reject the predicted result (*discrepant with evidence*) or the absence of sufficient evidence, such as targeted or RNA sequencing, to completely exclude the prediction (*discrepant but inconclusive*). ‘Represented samples’: diagnoses where the subtype was represented by a methylation class. ‘Unrepresented samples’: diagnoses not represented in the DKFZ Classifier. (C) The estimated tumour purity is higher in predicted (calibrated score ≥0.9) cases compared to cases not receiving a prediction (*p* = 0.008, Student's *t*‐test).

As the DKFZ Classifier v12 does not have a methylation class representing every soft tissue and bone tumour type, our data set was divided into two groups: those with a diagnosis represented by a methylation class in the DKFZ Classifier (*n* = 820, henceforth referred to as our ‘core validation samples’) and those samples with no methylation class corresponding to our histological diagnoses (*n* = 163, ‘unrepresented samples’). We analysed the classifier performance on both the entire data set (Figure 1A) and on the core validation samples (Figure 1B). We included tumour subtypes not included in the original DKFZ Classifier as it was important to test how the classifier handled subtypes for which it was not trained originally.

### Assessing DKFZ Classifier predictions

Based on the results of the DKFZ Sarcoma Classifier and any additional pathology review, all cases were divided into four main groups (Figure 1): (1) *Concordant*: samples predicted to a methylation class matching the histological diagnosis or which led to a revised diagnosis. (2) *Discrepant*: where the predicted methylation class did not match the original histological diagnosis, and following review there was either sufficient evidence to reject the predicted result (*discrepant with evidence*) or the absence of sufficient evidence such as RNA sequencing to exclude completely the prediction (*discrepant but inconclusive*). (3) *False negatives*: samples belonging to the core validation cohort that did not receive a prediction and (4) *True negatives*: samples not represented in the DKFZ Classifier reference set that did not receive a prediction. Evidence employed to support or reject a prediction included characteristic molecular alterations, pathognomonic histological features, anatomical location, and, where available, characteristic radiological features. A detailed description of the investigation of discrepant results, tumour purity estimation, and the statistical analysis performed can be found in Supplementary materials and methods.

## Results

### Predictions are concordant with the histological diagnosis in 88% of cases

The performance of the DKFZ Classifier was evaluated against a total of 986 of our samples (935 tumour and 51 controls), of which 3 tumour samples failed the quality control employed in the DKFZ Classifier [5] and were excluded from further analysis (Figure 1A and supplementary material, Table S1).

Analysis of our entire cohort using the DKFZ Classifier revealed that 541 of 983 (55%) samples were predicted to belong to one of the defined methylation classes, 83% (451/541) of which were predicted to the methylation class corresponding to their histological diagnosis (concordant). This included six samples where the original histological diagnosis following pathology review was changed in favour of the predicted methylation class (Table 1 [14, 15] and Figure 1A). Conversely, 17% of predictions (90/541 samples) were found to be discrepant: 44 of these cases were categorised as discrepant with evidence following further investigations which substantiated the original histological diagnosis and/or rejected the predicted diagnosis. The other 46 cases were classified as discrepant but inconclusive as the histology was not definitive for the diagnosis, and sufficient evidence was unavailable to exclude the prediction. The remaining 45% (442/983) of samples did not reach the threshold score, i.e. the DKFZ Classifier was unable to recognise these tumours (Figure 1A).

**Table 1:** Revised diagnoses based on DKFZ Classifier results.

Case	Original histological diagnosis	Predicted DKFZ methylation class	Prediction score	Histology review/IHC validation	Molecular validation	Revised diagnosis
166	Myxofibrosarcoma	MPNST class	0.944	H3K27me3 negative	—	MPNST
287	USARC	DFSP class	0.999	Positive: SMA (focal), CD34 (focal) Negative: S100, desmin, MNF116	*COL2A1‐PDGFB* rearrangement (FISH)	High‐grade transformation of a dermatofibrosarcoma protuberans
884	Osteosarcoma	SEF class	1.000	Positive: MUC4, INI1, CD99	—	Sclerosing epithelioid fibrosarcoma of bone
964	MPNST	SBRCT_CIC class	0.999	—	*CIC‐DUX4* rearrangement (FISH)	*CIC*‐rearranged sarcoma^†^
965	MPNST	SBRCT_BCOR class	1.000	BCOR positive	*BCOR‐CCNB3* rearrangement (PCR)	*BCOR*‐rearranged sarcoma*
254	USARC	Leiomyosarcoma class	0.998	Pleomorphic spindle cell tumour with areas of smooth muscle differentiation Positive: SMA and (focal) caldesmon	—	Leiomyosarcoma (pleomorphic)

All cases were originally diagnosed between 2008 and 2012.

DFSP, dermatofibrosarcoma protuberans; FISH, fluorescence *in situ* hybridization; IHC, immunohistochemistry; PCR, polymerase chain reaction; SBRCT_CIC, small blue round cell tumour with *CIC* alteration; SBRCT_BCOR, small blue round cell tumour with *BCOR* alteration; SEF, sclerosing epithelioid fibrosarcoma; SMA, smooth muscle actin.

^*^ Recently defined sarcomas which at the time of the original diagnosis were yet to be discovered [14].

^†^ Recently defined sarcomas which at the time of the original diagnosis were not widely recognised as distinct entities [15].

We next limited our analysis to the core validation set (Figure 1B); this resulted in the DKFZ Classifier providing a prediction for 61% (499/820) of samples. Eighty‐eight percent (440/499) of these predictions were concordant, including six cases where we revised our diagnoses. The classifier score for 321 samples, representing 39% of the core validation samples, was below the 0.9 threshold and therefore these did not receive a prediction despite having a representative methylation class for their histological subtype.

### Methylation profiles may provide misleading diagnoses

The DKFZ Classifier results for 59 of our core validation samples were discrepant with our histological diagnoses (Figure 1B). Sufficient evidence to support the original diagnosis and reject the classifier prediction was obtained for 31 of these cases, whereas 28 cases were classified as discrepant but inconclusive (Figure 1B and supplementary material, Table S1).

We observed that some histological subtypes were more frequently misclassified to specific methylation classes, including high‐grade osteosarcomas (OS), 11 (5% of OS) of which were assigned to the undifferentiated sarcoma (‘USARC’) class despite having typical radiological imaging of a primary bone tumour and with unequivocal morphological evidence of osteoid deposition. Similarly, five dedifferentiated chondrosarcomas (CHSs) with osteosarcomatous differentiation (38% of dedifferentiated CHS) were predicted to the OS high‐grade methylation class (‘OS_HG’). In all cases, the tumours also contained a sharply defined well‐differentiated cartilaginous component, the diagnostic hallmark of a dedifferentiated CHS, and two cases harboured *IDH1* mutations (cases 360 and 382; see supplementary material, Table S1).

The DKFZ Classifier includes a ‘SARC_MPNST_LIKE’ methylation class, a subset of malignant peripheral nerve sheath tumours (MPNST), which retains expression of H3K27me3. Of our 79 MPNSTs submitted to the classifier, 32 received a prediction. Of these, 19 exhibited the classical histopathological features of MPNST and loss of H3K27me3 and were correctly predicted to the ‘MPNST’ methylation class. One sample with retained H3K27me3 (case 948) and one sample with H3K27me3 loss were classified as SARC_MPNST_LIKE (case 723). The remaining 11 cases were predicted to various methylation classes, most commonly the USARC class but also the synovial sarcoma and *CIC*‐rearranged sarcoma classes. Following a thorough review, these 11 cases were still considered to represent MPNST with retained H3K27me3 expression, a finding now well recognised [8, 16]. Evidence to support this diagnosis of MPNST was available for 4 of 11 cases, with two tumours arising in patients with germline *NF1* alterations (cases 224 and 798) and two others associated with deep‐sited nerves (cases 811 and 960). Classifier predictions for the remaining 7 of 11 MPNSTs were deemed discrepant but inconclusive (see supplementary material, Table S1).

### Tumour purity correlates with calibrated score but not prediction accuracy

To determine if tumour purity accounted for the failure of the DKFZ Classifier to provide accurate prediction methylation classes, we estimated the tumour purity in all samples using the ‘RF‐purity’ package [17] (see Supplementary materials and methods). We did not observe a difference in the tumour purity of samples given a prediction matching their respective histological diagnoses compared to those given predictions deemed to be incorrect (*p* = 0.36, Student's *t*‐test; Figure 1C and supplementary material, Figure S1). However, predicted samples demonstrated higher tumour purity than those not receiving a prediction (*p* = 0.008, Student's *t*‐test; Figure 1C). We next examined the 321 of 820 (39%) samples that did not receive a prediction due to insufficient calibrated scores and found that 45% (144/321 samples) of those cases were still assigned to the correct methylation class (see supplementary material, Table S1). Furthermore, the estimated tumour purity was higher in those samples with scores below threshold, but still correctly recognised by the classifier, compared to those with scores below threshold with the classifier providing an incorrect result (*p* = 8e‐07, Student's *t*‐test; see supplementary material, Figure S2). Notably, the tumour purity across our samples (mean: 44%, range: 26–69%) was comparable to that of the DKFZ validation samples (mean: 47%, range: 26–71%) [5].

Based on our tumour purity results, we investigated if a tumour purity filtering step could increase the fraction of cases being assigned a diagnostic prediction. A tumour purity cut‐off value of 0.4 was applied, and although it raised the median number of samples that were accurately classified for several subtypes, this resulted in a lower number of samples that received a prediction; furthermore, this filtering step lowered the accuracy for specific subtypes including epithelioid sarcoma and chondroblastoma (see supplementary material, Table S2 and Figure S3). We also tested whether a reduced calibrated score threshold of 0.85 would improve the DKFZ Classifier performance on our data set. While this resulted in an increased number of samples receiving a classifier prediction (64% at 0.85 threshold versus 61% at 0.9), there was no significant improvement in the proportion of samples that were predicted correctly (87% of core validation cohort at 0.85 threshold versus 88% at 0.9 threshold) (see supplementary material, Table S3). We noted that DNA extracted from formalin‐fixed paraffin‐embedded (FFPE) samples (*n* = 320) obtained lower calibrated scores than those obtained from fresh frozen tissue samples (*n* = 645) (*p* = 2e‐06, Student's *t*‐test; see supplementary material, Figure S4). This translated into a lower proportion of FFPE samples being given a prediction compared to frozen samples (45 versus 59%, *p* = 0.02, Pearson's chi‐squared test). However, this difference was not related to the estimated tumour purity (*p* = 0.12, Student's *t*‐test).

### Classifier performance varies across tumour types

It was noted that specific tumour subtypes received a prediction to the methylation class matching their histological diagnosis more often than others (Table 2 and supplementary material, Figure S5 and Table S4); these included mesenchymal CHSs (88% sensitivity), chordomas (85% sensitivity), and fibrous dysplasia (83% sensitivity) in our data set. Conversely, clear cell CHSs, neurofibromas, and MPNSTs represented the tumour types with the lowest number of samples predicted to the correct methylation class (0, 14, and 25% sensitivity, respectively). The low sensitivity achieved for these latter two entities was largely due to the low proportion of cases from these tumour types gaining a classifier score above threshold. Notably, these differences in sensitivity across tumour types were independent of tumour purity (see supplementary material, Figures S6 and S7; *p* = 0.13, Spearman's rank correlation).

**Table 2:** Overview of the main included sarcoma subtypes and controls.

Sarcoma subtype/group	Included in DKFZ Classifier	Proportion predicted to correct methylation class (%)
Adamantinoma (*n* = 19)	No	—
Alveolar soft part sarcoma (*n* = 11)	Yes	81.8
Aneurysmal bone cyst (*n* = 13)	No	—
Angiosarcoma (*n* = 10)	Yes	50.0
Blood controls (*n* = 20)	Yes	90.0
Chondroblastoma (*n* = 17)	Yes	58.8
Chondromyxoid fibroma (*n* = 14)	No	—
CHS, conventional (*n* = 97)	Yes	36.1
CHS, clear cell (*n* = 7)	Yes	14.3
CHS, mesenchymal (*n* = 8)	Yes	87.5
Chordoma (*n* = 88)	Yes	85.2
Epithelioid sarcoma (*n* = 8)	Yes	62.5
Fibrous dysplasia (*n* = 6)	Yes	83.3
Giant cell tumour of bone (*n* = 57)	Yes	70.2
Leiomyosarcoma (*n* = 6)	Yes	50.0
MPNST (*n* = 79)	Yes	25.3
Myxofibrosarcoma (*n* = 56)	Yes	42.9
Neurofibroma (*n* = 6)	Yes	16.7
Non‐ossifying fibroma (*n* = 13)	No	—
Normal bone (*n* = 9)	No	—
Normal muscle (*n* = 5)	Yes	80.0
Normal tissue, NOS (*n* = 15)	Yes	60.0
Osteoblastoma (*n* = 12)	Yes	75.0
OS, high‐grade central (*n* = 198)	Yes	55.1
OS, parosteal, (*n* = 25)	No	—
OS, extraskeletal (*n* = 18)	No	—
PEComa (*n* = 8)	No	—
Phosphaturic mesenchymal tumour (*n* = 7)	No	—
Pleomorphic liposarcoma (*n* = 14)	Yes	28.6
Rhabdomyosarcoma (*n* = 7)	Yes	57.1
Undifferentiated pleomorphic sarcoma (*n* = 79)	Yes	51.9

Overview of the sarcoma subtypes with more than five samples included in this study. The full list of samples and subtypes included can be found in supplementary material, Table S1. Subtypes not represented in the DKFZ Classifier were included to demonstrate how the classifier handled subtypes for which it was not yet trained. Dedifferentiated CHSs are included under the conventional CHS category.

NOS, not otherwise specified; PEComa, perivascular epithelioid cell tumour; CHS, chondrosarcoma.

Analysis of our conventional chondrosarcomas (CHS) resulted in 56 of 98 samples not being predicted to a methylation class, which was partly accounted for by the DKFZ Classifier providing four different classes for these tumours based on *IDH1/2* mutational status. For 15 of 56 tumours, the classifier could not confidently select one of these four classes, whereas by combining the classifier score given to each of these four classes (a ‘CHS family score’) these cases would have been predicted to the correct ‘methylation family’ (see supplementary material, Table S1), an observation also noted in the validation performed by Koelsche *et al* of the DKFZ Classifier [5]. We also identified a non‐random distribution of the methylation chip type (450K versus EPIC arrays) associated with the different tumour types (see supplementary material, Figure S8); however, an equal proportion (61%) of core samples on both chip types received a score above threshold.

### Classification of pleomorphic sarcomas is rarely improved by methylation profiling

Samples from 79 USARCs of bone and soft tissue were included in this study (see supplementary material, Table S1). In line with the WHO criteria, these tumours were defined by their histological features together with an absence of immunohistochemical or molecular features indicative of a distinct entity or cell lineage [18, 19]. Forty‐six (58%) of these samples received a classifier prediction, with the majority (41/46) assigned to the USARC methylation class and 1 sample to the SARC_MPNST_LIKE methylation class. Seven samples were assigned to classes representing specific sarcoma subtypes. Following review, two of these predictions led to a revised diagnosis (Table 1; cases 254 and 287); one was categorised as discrepant but inconclusive and the others were rejected based on histological and molecular evidence. Conversely, 18 samples of high‐grade sarcomas with histological and/or molecular features indicative of distinct histotypes were given a prediction to the USARC methylation class. These predictions could be explained on the basis that the samples represented a dedifferentiated component of a particular sarcoma subtype, such as a chordoma (case 758), or showed a significant degree of pleomorphism while otherwise exhibiting characteristic histological features in keeping with our original histological diagnosis, for instance high‐grade conventional OS (see supplementary material, Table S1).

### A quarter of tumours not represented in the DKFZ Classifier receive a prediction

**Figure 2:**
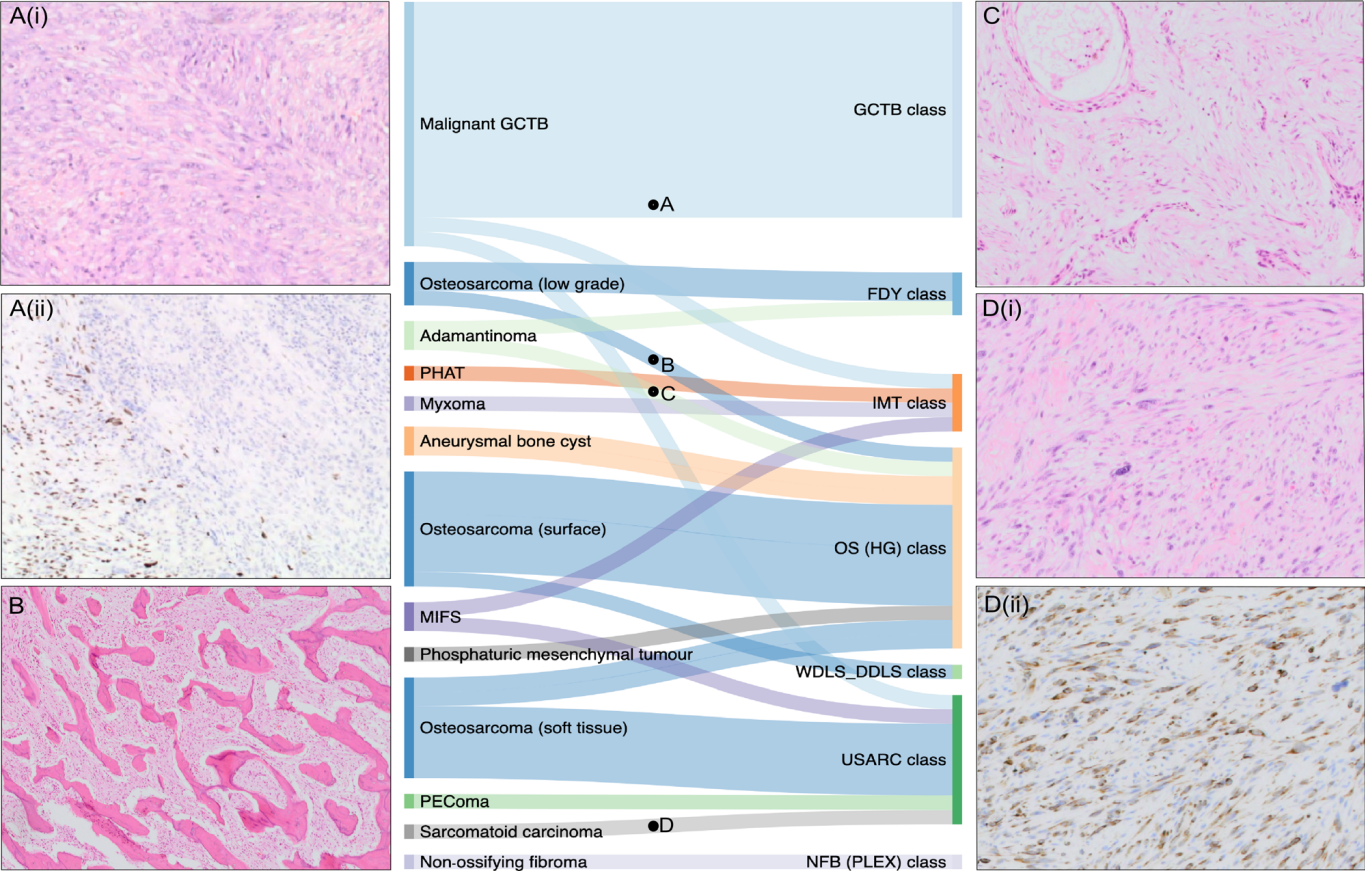
Sankey plot showing the classifier predictions of samples with a subtype not represented in the current version (v12) of the ‘DKFZ sarcoma Classifier’. (A) Case 826, (i) Haematoxylin and eosin (H&E) demonstrating high‐grade spindle cell areas of a malignant GCTB with (ii) focal loss of H3F3A G34W expression on immunohistochemistry. (B) Case 120, H&E showing typical bony trabeculae within a low‐grade parosteal OS. (C) Case 828, H&E showing a spindle cell lesion with scattered squamous islands characteristic of an adamantinoma. (D) Case 311, (i) H&E of high‐grade spindle cell lesion in a patient with a background of breast carcinoma; (ii) the lesion showed widespread CAM5.2 immunopositivity and was subsequently diagnosed as a metastatic focus. FDY, fibrous dysplasia; HG, high grade; IMT, inflammatory myofibroblastic tumour; MIFS, myxoinflammatory fibroblastic sarcoma; NFB(Plex), plexiform neurofibroma; PEComa, perivascular epithelioid cell tumour; PHAT, pleomorphic hyalinising angiectatic tumour; WDLS_DDLS, well‐differentiated liposarcoma/dedifferentiated liposarcoma.

One hundred and sixty‐three samples of our sample set, representing 21 distinct tumour subtypes (19 soft tissue and bone tumours, 2 carcinomas), were not represented in the DKFZ Classifier v12 (see supplementary material, Table S1 and Figure S9). Twenty‐six percent (42/163) of these received a prediction (score >0.9 threshold) to one of the existing methylation classes (Figure 2). Samples from 11 malignant giant cell tumour of bones (GCTBs) clustered with conventional GCTBs, a finding explained on the basis of the shared cell of origin and the absence of a specific class for malignant GCTB in the DKFZ Classifier. None of the predictions of the remaining 31 samples warranted a revised diagnosis, although it was noted that many of these samples were assigned to a methylation class which represented a closely related tumour subtype for which the classifier had been trained (Figure 2). For example, parosteal and periosteal OS were classified under the OS_HG methylation class.

## Discussion

In this study, we assessed methylation data from 983 samples by employing the DKFZ methylation‐based sarcoma classifier, the most comprehensive DNA methylation‐based tool published to date on these rare tumours [5]. We found that 61% of our samples for which the classifier had been trained (the core validation set) were given a prediction, and that our histological diagnoses were concordant with the assigned methylation class in 88% of these samples. The reason for the greater number of samples failing to meet the calibrated score threshold in our data set (39%) compared to those in the original validation study (25%) is unclear. Tumour purity, a recognised potential confounder in the analysis of DNA methylation analysis [20], did not account for the difference. The non‐random distribution of sarcoma types across the two methylation chip types was similarly unlikely to explain these differences, as the classifier reference set includes samples run on both the 450K and the EPIC chips and a batch adjustment for chip type is performed as part of the classifier pipeline. Furthermore, we found a similar proportion of cases on both chip types receiving classification. Interestingly, a smaller proportion of classified samples was also noted when the DKFZ brain classifier [3] was validated on external data sets [4, 21], indicating that this may be related to institution‐specific factors and the greater experience that the DKFZ group has in sample preparation for methylation analysis.

From a diagnostic perspective, 9% of samples would have been misclassified in the original DKFZ validation data set [5]. In this current study, the proportion of misclassified samples was 12% (6% with definitive molecular evidence and 6% with strong histological/clinical evidence but without definitive molecular evidence). This may partly be explained by the Heidelberg Sarcoma Classifier reference set being composed of ‘classical’ cases with confirmed pathognomonic alterations for all entities characterised by such a feature. The classifier was therefore trained on a relatively narrow spectrum of cases for each sarcoma subtype compared to those in our data set, resulting in a greater number of discrepant results in our study. The error rate reported for the two validation cohorts demonstrates that there is room to optimise the Sarcoma Classifier to the diagnostic level precision of the brain tumour classifier [3]. Conversely, the percentage of predictions which resulted in correction of a diagnosis was 9% in the original DKFZ validation study (29/322 predicted samples) and 1.2% in our study (6/499 predicted samples). This may reflect that the original validation cohort included samples from a number of centres, while in this study cases were submitted from a single specialist sarcoma centre.

We noted that the classifier performed best when diagnosing mesenchymal CHAs, chordomas, and fibrous dysplasia, while lower overall rates of classification and accuracy were observed amongst sarcoma subtypes known to be genomically complex and exhibit high levels of tumour heterogeneity, including high‐grade OS [22, 23], myxofibrosarcomas [24], pleomorphic liposarcomas [25], and MPNSTs [26]. It is noteworthy that Koelsche *et al* found that many of these tumours, which pathologists distinguish on the basis of histology alone, formed a single methylation cluster [5]. Specifically, myxofibrosarcomas, undifferentiated pleomorphic sarcoma, and pleomorphic liposarcoma formed a USARC cluster, supporting the concept that they may represent the same disease, a concept also suggested by existing genomic evidence [9, 11]. The overlapping methylation profiles of these different sarcoma subtypes is consistent with the concept that aberrant DNA methylation patterns reflect the cell of origin of the tumour [27]. However, this begs an explanation for their distinctive histological features [18]. Could it be that these tumours arise from cells at marginally different states of commitment which cannot be distinguished by their methylation profile, at least when using the Illumina Infinium Methylation arrays, but is still reflected in their morphology?

The diagnosis of MPNST has always been challenging because of its histological variation and the absence of a surrogate marker for the biallelic loss of function of *NF1*. Loss of H3K27me3 expression, largely specific to high‐grade MPNST, recently provided a valuable marker for this disease [28]; however, evidence has accumulated that a significant proportion of MPNSTs do not exhibit this molecular alteration [8, 16]. It was therefore interesting that the DKFZ group identified a methylation cluster of MPNSTs which retained expression of H3K27me3 (SARC_MPNST_LIKE) [10]. Our data did not fully reproduce this finding leaving unresolved challenges around the classification of MPNST. Ongoing comprehensive multi‐omic studies may help provide answers [12, 29].

Tumour subtypes unrepresented in the DKFZ Classifier should not receive predictions above threshold, but 26% of samples did obtain a classification. It is noteworthy that these samples did not seem to be predicted to random classes but in most instances were classified as closely related tumour entities. It is interesting to speculate that this is due to the tumours sharing a cell of origin or other biological relationships which are worthy of further study. Nevertheless, these predictions have clinical implications which could be detrimental for patients. For instance, distinguishing parosteal OS from high‐grade OS has important management consequences. Expanding the spectrum of diagnoses in the classifier may allow such subtypes to be distinguished, but this will be a major task because of the large number of histological mimics of sarcoma.

By asking rigorous biological and clinically relevant questions, the classification of disease has the potential to provide novel prognostic and predictive biomarkers as well as identify therapeutic targets. Studies of the methylation profiles of GCTBs, dedifferentiated liposarcoma, MPNST, and CHA serve as examples [6, 7, 8, 11]. In the DKFZ Classifier, benign and malignant GCTBs cluster together; however, directed analysis of methylation data of these subtypes not only allowed them to be separated, but also implicated *CCND1* as a likely cancer driver gene in the malignant transformation of GCTB [7]. Second, The Cancer Genome Atlas analysis of soft tissue sarcomas demonstrated that dedifferentiated liposarcoma, conventionally classified by the presence of *MDM2* amplification, formed two methylation groups which corresponded to significantly different clinical outcomes [11]. Refinement of the DKFZ Classifier should allow for these clinically significant tumour subgroups to be distinguished.

The challenges faced in determining the reasons behind incorrect and failed classifications represent an important limitation of machine‐learning approaches, including random forest classifiers [30] and has resulted in considerable debate and distrust with respect to the use of ‘black box’‐type and machine‐learning models in clinical applications [31]. This has stimulated the development of new approaches capable of explaining various features employed in the decision‐making processes which provide a classifier result [30, 32]. However, concerns remain whether these *post hoc* interpretability models are the correct approach for clinical use [33].

A limitation of our study is the lack of comprehensive multi‐omic molecular interrogation for all cases, which potentially could have provided clarification of the nature of those tumours categorised as discrepant but inconclusive as well as further evidence for classification of those tumours categorised as discrepant with evidence where the evidence was based on morphology and immunohistochemistry grounds. However, as a minimum, diagnoses were reached using rigorous tests employed in the current standard of care clinical setting. Classification of disease is an ongoing process and will continue to be modified to reflect what we know about clinical outcome and response to therapies. We believe that the current version of the classifier is most valuable at a research level and should lead to a greater understanding of the pathogenesis of sarcoma. However, although, in its current form, it can provide supportive evidence and may prompt a diagnosis, these results still require validation. With additional work, the DKFZ has the potential to contribute to diagnostic pathology in a more routine setting.

## Author contributions statement

IL, AMF and AVD were responsible for the study design. IL, AMR, SDN, DSc, DSt, MS, CK and AMF collected data. IL, SDN, AMF and SB analysed and interpreted data. SDN, AMF, RT, DL and FA were responsible for pathology review. IL, AMF, SDN and SB wrote the manuscript, with contributions from NP and FA. All the authors reviewed and edited the paper.

## Supporting information


Supplementary materials and methods

**Figure S1.** No difference in estimated tumour purity between samples predicted correctly versus those predicted incorrectly in the ‘core validation cohort’
**Figure S2.** The tumour purity is higher in samples correctly classified despite having a calibration score below threshold
**Figure S3.** Test of tumour filtering step
**Figure S4.** FFPE samples obtain a lower calibrated classifier score than fresh frozen tissue samples
**Figure S5.** Core validation set – results by tumour type
**Figure S6.** Proportion of cases per subtype predicted to the correct methylation class is independent of tumour purity
**Figure S7.** Correlation plot showing no clear correlation between estimated tumour purity and the proportion of cases predicted correctly (sensitivity) per subtype
**Figure S8.** Non‐random distribution of the methylation chip type (450K versus EPIC arrays) associated with the different tumour types
**Figure S9.** t‐distributed stochastic neighbour embedding (t‐SNE) showing the clustering of the unrepresented samples

**Table S1.** Overview of RNOH samples
**Table S2.** Inclusion of estimate tumour purity filtering step before validating the performance of the classifier
**Table S3.** Testing a classifier score threshold cut‐off = 0.85
**Table S4.** Sensitivity and precision of the different methylation classes (core validation set)
**Table S5.** Proportion of samples predicted to the correct methylation class (DKFZ validation and RNOH cohorts, common tumour types)

## Data Availability

Raw methylation array data are available via the ArrayExpress database at EMBL‐EBI (www.ebi.ac.uk/arrayexpress) under accession number E‐MTAB‐9875.
