# Supplementary material for: DNA methylation‐based profiling of bone and soft tissue tumours: a validation study of the ‘DKFZ Sarcoma Classifier’
Source: J Pathol Clin Res. 2021 May 5;7(4):350–60. doi: 10.1002/cjp2.215 (PMC8185366; doi:10.1002/cjp2.215)
Supplement: Supplementary file 1 — Supplementary materials and methods [file CJP2-7-350-s003.docx]

**DNA methylation-based profiling of bone and soft tissue tumours: a validation study of the ‘DKFZ sarcoma Classifier’**

I Lyskjær *et al*. *J Pathol Clin Res* DOI: 10.1002/cjp2.215

**Supplementary Materials and Methods**

Reference numbers refer to the list in the main paper

**DNA methylation profiling**

500 ng of DNA from fresh frozen (FT) or formalin-fixed paraffin-embedded (FFPE) tumour samples were bisulphite converted using the Zymo EZ DNA methylation Gold kit (Zymo Research Corp. Irvine, USA) before hybridisation to the Infinium HumanMethylation450 or EPIC beadchip arrays (Illumina, San Diego, CA) by UCL Genomics. All bisulphite-converted FFPE samples were restored with the Infinium FFPE DNA Restore kit (Illumina).

**Investigation of discrepant results**

All cases where the Classifier result was discrepant with the original histological diagnosis were reviewed by specialist pathologists, including histology slides, and available radiology, immunohistochemical and molecular results. Definitive evidence to support a diagnosis included (where applicable): i) classical histological features, e.g. osteoid formation in high grade bone tumour (osteosarcoma), juxtaposed low grade cartilaginous regions and high grade dedifferentiated tumour (dedifferentiated chondrosarcoma); ii) characteristic immunohistochemical profiles, e.g. brachyury expression (chordoma), H3.3 G34W expression (giant cell tumour of bone); iii) radiological features/anatomical location, e.g. tumour arising from nerve (MPNST, neurofibroma), tumour arising from bone surface (surface osteosarcoma); iv) recurrent molecular alterations, e.g. *MDM2* amplification (parosteal osteosarcoma and well differentiated/dedifferentiated liposarcoma), *IDH1/IDH2* mutations (conventional and dedifferentiated chondrosarcoma), *BCOR* rearrangement (FISH, *BCOR*- rearranged sarcoma) and v) pathognomonic clinical history, e.g. germline NF1 alteration (MPNST).

Additional tests were performed on an individual case basis to provide evidence to support or refute the Classifier prediction as follows:

Immunohistochemical (IHC) testing was performed in accordance with standardised procedures [34] using the following antibodies: anti-SMARCB1 (INI1/BAF47; clone 25/BAF47, 1/400, BD Biosciences, UK), anti-histone 3.3 G34W (H3.3G34W; clone RM263, 1/1500, RevMAb Biosciences, USA), Tri-Methyl-Histone H3 (Lys27) (H3K27me3; clone C36B11, Cell Signalling Technology, London, UK), anti-mucin 4 (MUC-4; clone 8G7, 1/400, Santa Cruz Biotechnology, USA), anti-BCOR (BCoR, clone C-10, 1/150, Santa Cruz Biotechnology, USA), anti-CD99 (clone PCB1, 1/100, Leica Microsystems, UK).

Interphase fluorescence in situ hybridisation (FISH) was performed as previously described [34] using commercially available dual colour probes: Agilent (Agilent, Santa Clara, USA) SureFISH CIC break-apart probe (CIC rearranged sarcoma; SBRCT (CIC) methylation class), Vysis (Abbott Laboratories, Illinois, USA) MDM2 CEP 12 and Zytolight (Zytovision, Bremerhaven, Germany) SPEC MDM2/CEN 12 (well differentiated/dedifferentiated Liposarcoma; WDLS/DDLS methylation class), Vysis SS18 break-apart probe (synovial sarcoma; SYSA methylation class), Zytolight SPEC USP6 break-apart probe (aneurysmal bone cyst) and Zytolight SPEC COL1A1-PDGFB fusion probe (dermatofibrosarcoma protuberans; DFSP methylation class). Reverse transcription-polymerase chain reaction (RT-PCR) was performed to detect BCOR-CCNB3 fusions (BCOR-rearranged sarcoma; SBRCT (BCOR) methylation class) using Eurofins Genomics (Louisville, USA) primers BCOR-exon15/CCNB3-exon5 (BCOR Reference Sequence: NM_001123383.1, primer sequence GGCTCCACCCCAGTGATCT, CCNB3 - Reference Sequence: NM_033031.2, primer sequence GGGTGTTTTGGAGGTGGTGGAT).

**Tumour purity** **estimation and statistical analysis**

The estimated tumour purity was computed for all cases using the ‘ABSOLUTE’ method in the ‘RF_Purify’ R package [17]. No statistical methods were used to predetermine sample size. All data analysis was done in R, version 3.6.1 [35]. Plots were generated using the following R packages: ggplot2 and ggpubr. Sankey plot was generated using SankeyMATIC (Beta) web-based tool (http://sankeymatic.com/build/). P-values were determined using Student’s t-test or Spearman's rank correlation for analysis where the values were not normal distributed.

To examine the correlation between tumour cellularity and estimated tumour purity, a subset of 18 samples were chosen across four tumour types (conventional chondrosarcoma, high grade osteosarcoma, undifferentiated sarcoma and chordoma) and histological tumour cellularity was scored under the categories “low”, “low/moderate”, “moderate”, “moderate/high”, and “high”. Histological scoring was performed blinded to the estimated tumour purity result.

Statistical analysis of classifier performance (supplementary material, Table S4) was calculated as follows: Sensitivity = cases from histological subtype predicted correctly/total number of cases from histological subtype; Precision = cases from histological subtype predicted correctly/total number of cases from histological subtype receiving a prediction.
