# Supplementary material for: DNA methylation‐based profiling of bone and soft tissue tumours: a validation study of the ‘DKFZ Sarcoma Classifier’
Source: J Pathol Clin Res. 2021 May 5;7(4):350–60. doi: 10.1002/cjp2.215 (PMC8185366; doi:10.1002/cjp2.215)
Supplement: Supplementary file 2 — Figure S1. No difference in estimated tumour purity between samples predicted correctly versus those predicted incorrectly in the ‘core validation cohort’ Figure S2. The tumour purity is higher in samples correctly classified despite having a calibration score below threshold Figure S3. Test of tumour filtering step Figure S4. FFPE samples obtain a lower calibrated classifier score than fresh frozen tissue samples Figure S5. Core validation set – results by tumour type Figure S6. Proportion of cases per subtype predicted to the correct methylation class is independent of tumour purity Figure S7. Correlation plot showing no clear correlation between estimated tumour purity and the proportion of cases predicted correctly (sensitivity) per subtype Figure S8. Non‐random distribution of the methylation chip type (450K versus EPIC arrays) associated with the different tumour types Figure S9. t‐distributed stochastic neighbour embedding (t‐SNE) showing the clustering of the unrepresented samples [file CJP2-7-350-s002.docx]

**DNA methylation-based profiling of bone and soft tissue tumours: a validation study of the ‘DKFZ sarcoma Classifier’**

I Lyskjær *et al*. *J Pathol Clin Res* DOI: 10.1002/cjp2.215

**Supplementary Figures**


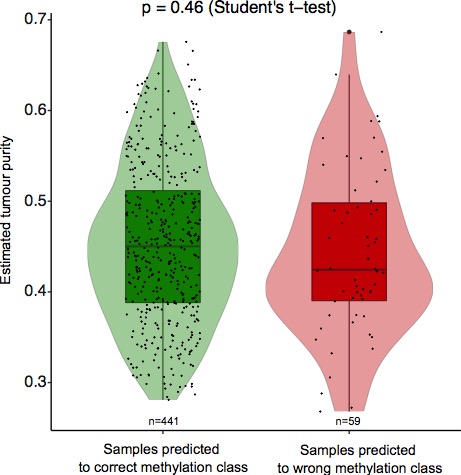


**Figure S1. No difference in estimated tumour purity between samples predicted correct versus those predicted incorrectly in the ‘core validation cohort’.** The number on the x-axis denotes the number of samples in the two groups.

**
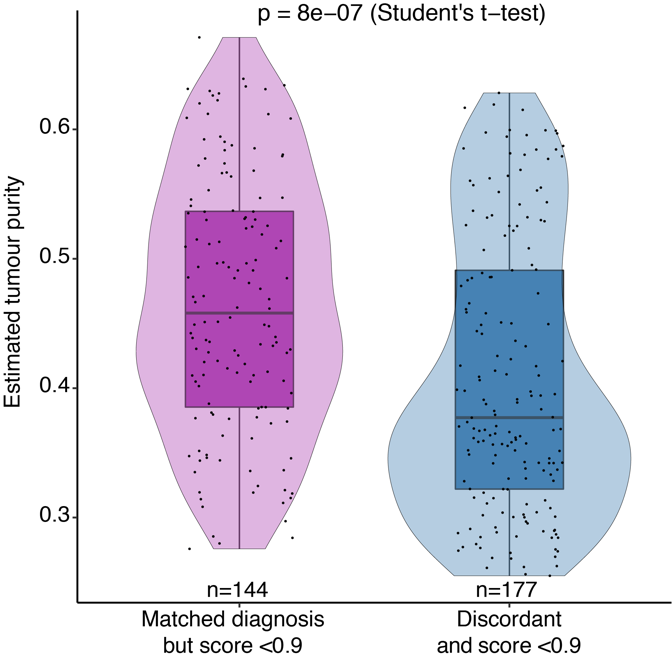
**

**Figure S2. The tumour purity is higher in samples correctly classified despite having a calibration score below threshold.** Estimated tumour purity plotted for non-predicted samples. For all cases not obtaining a prediction, the methylation class obtaining the highest score (but still below the 0.900 threshold) was evaluated to determine if they were still concordant with the original histological diagnosis (n=144) versus discordant (n=177).

**
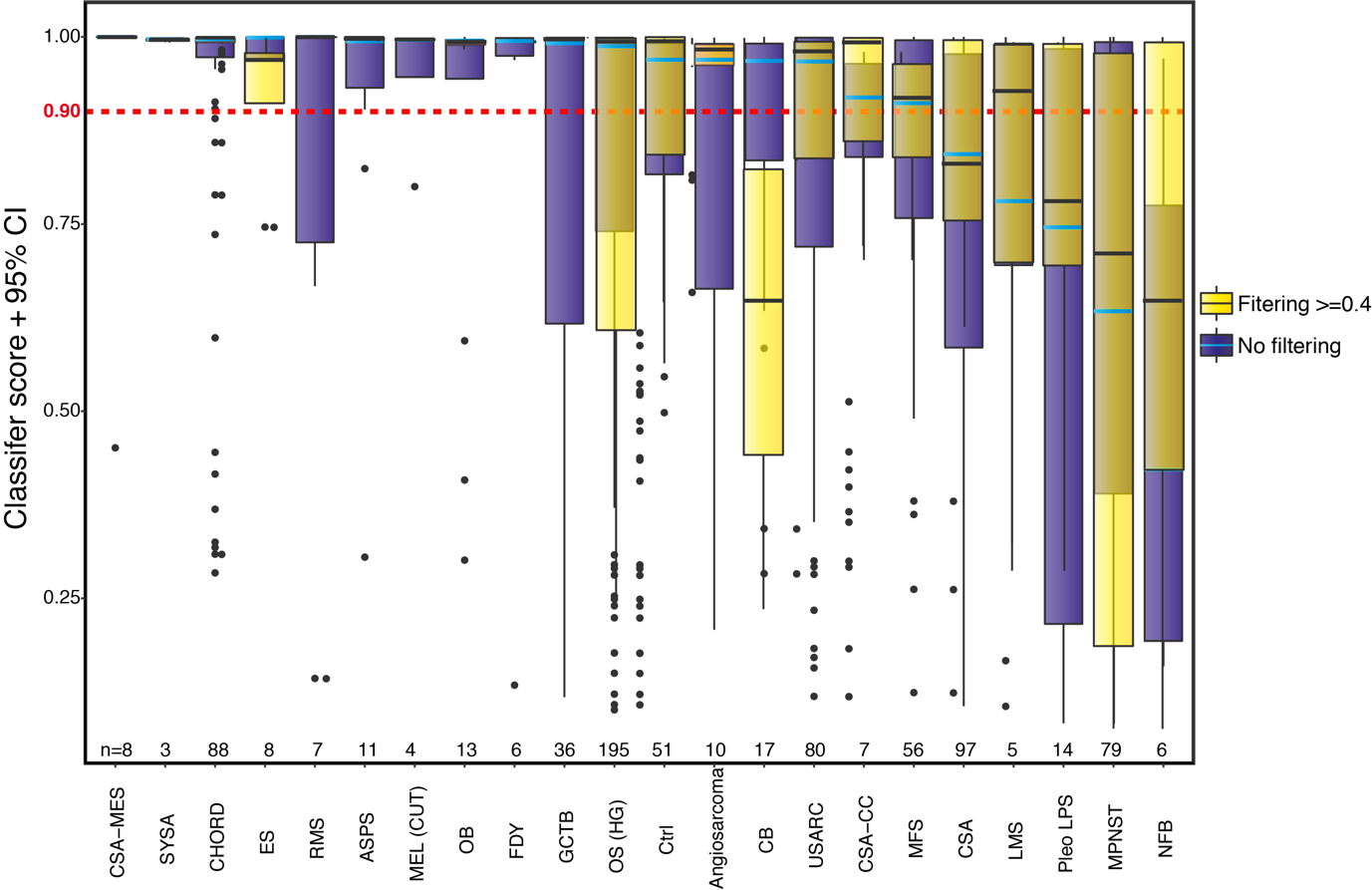
**

**Figure S3. Test of tumour filtering step.** The green bars indicate classifier scores after filtering for estimated tumour purity (>= 0.4), while purple bars are classifier scores without filtering. Medians are shown as vertical lines; blue is the median for non-filtered ones, while the black indicates the median after filtering. The red stippled line indicates a score leading to a successful prediction. Numbers at the bottom indicates the number of samples for the given subtype on the x-axis. Only subtypes with at least three samples are included.

CSA-MES: mesenchymal chondrosarcoma; SYSA: synovial sarcoma; CHORD: chordoma; ES: epithelioid sarcoma; ASPS: alveolar soft part sarcoma; MEL (CUT): cutaneous melanoma; OB: osteoblastoma; FDY: fibrous dysplasia, GCTB: Giant cell of Tumour Bone; OS (HG): high-grade osteosarcoma; Ctrl: control; CB: chondroblastoma; USARC: undifferentiated sarcoma; CSA-CC: clear cell chondrosarcoma; MFS: myxofibrosarcoma; CSA: chondrosarcoma; LMS: leiomyosarcoma, Pleo LPS: pleomorphic leiomyosarcoma, MPNST: malignant peripheral nerve sheath tumours; NFB: neurofibroma


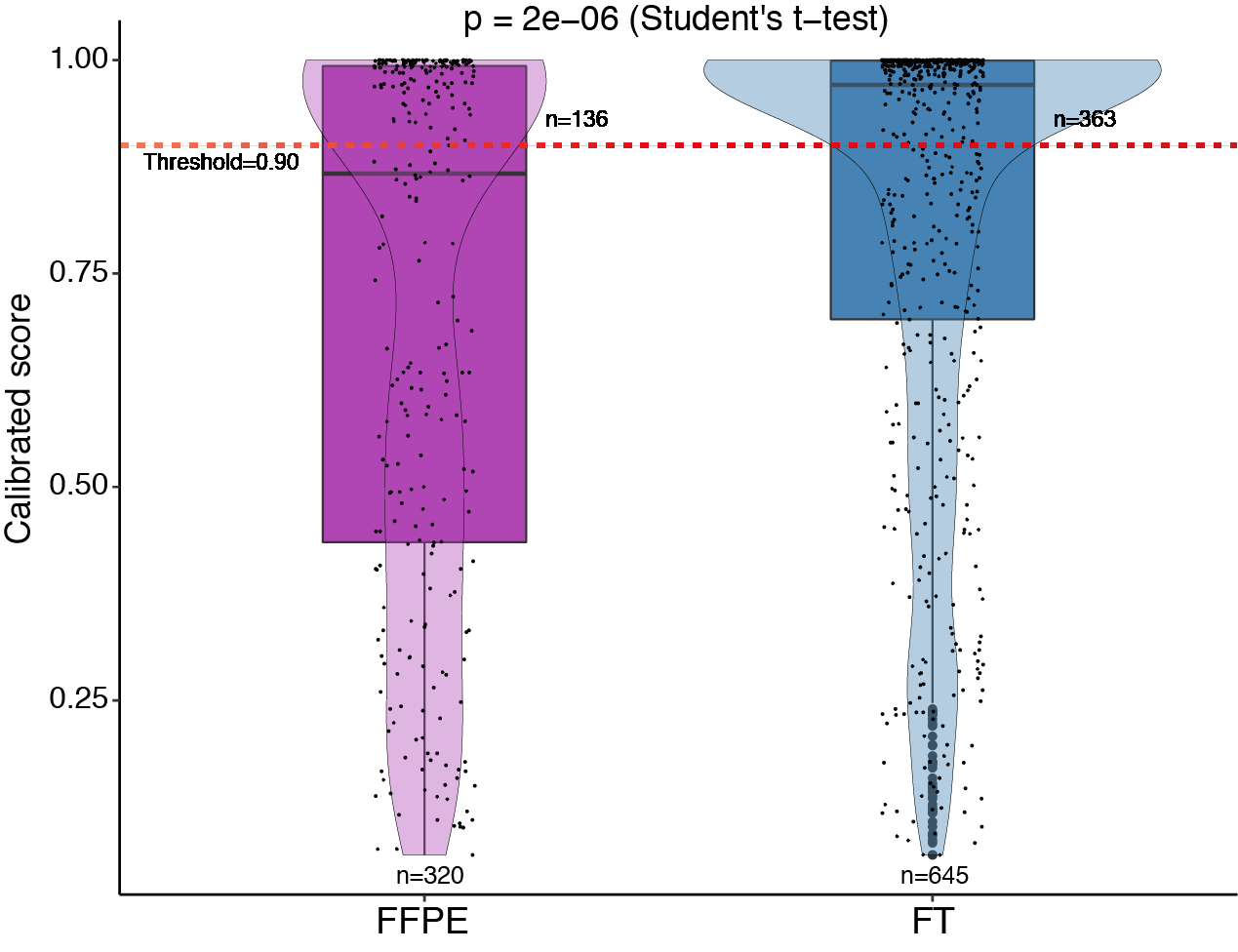


**Figure S4. Formalin-fixed paraffin-embedded samples obtain a lower calibrated classifier score than fresh frozen tissue samples.** The red dotted line denotes the classifier score threshold of 0.9 for successful prediction to a methylation class. The numbers above the red line indicate the number of samples with scores at or above the threshold. The numbers above the x-axis indicate the number of samples in the FFPE and FT group. FFPE: Formalin-fixed paraffin-embedded, FT: fresh frozen tissue.

**
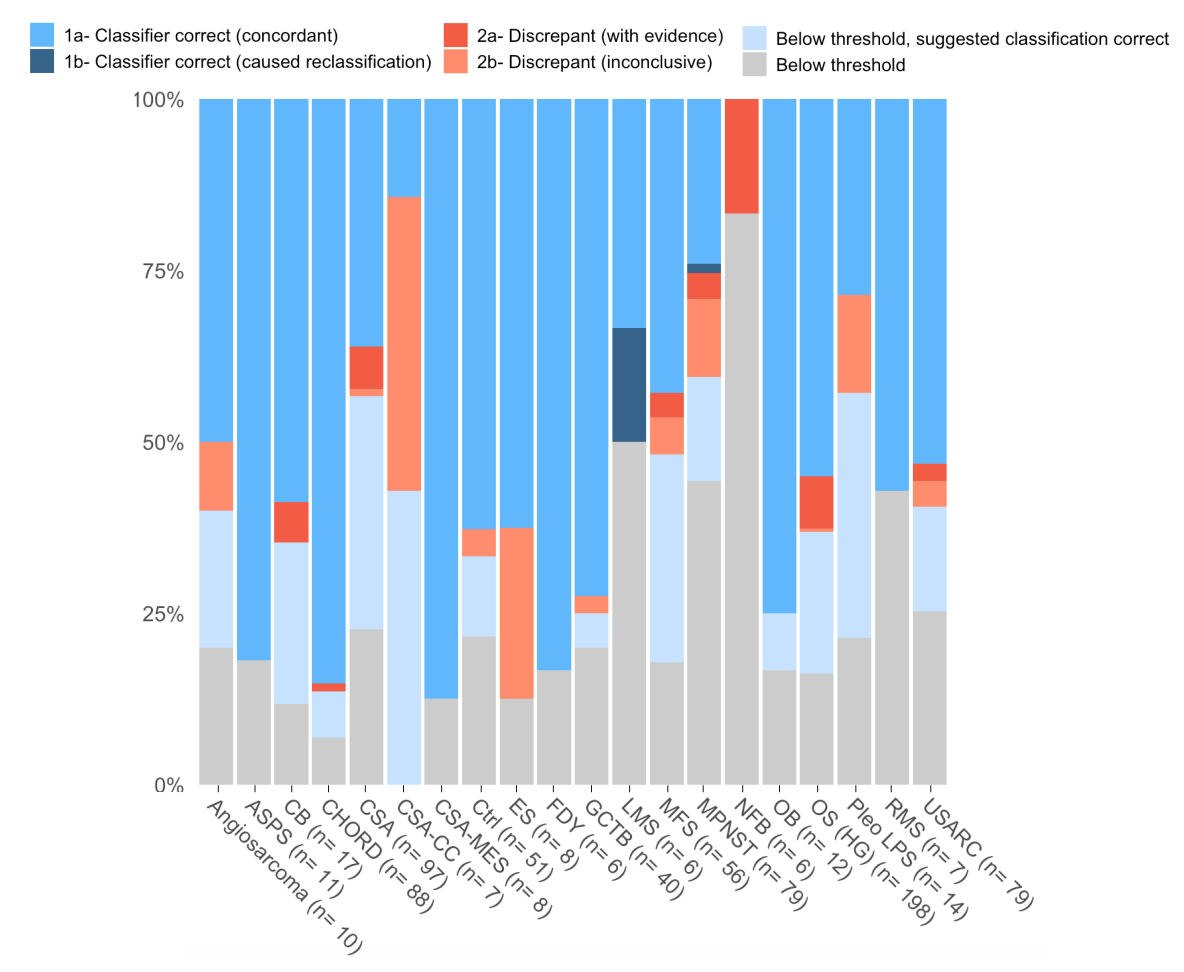
**

**Figure S5. Core validation set – results by tumour type.** The proportion of samples assigned to our result groups is demonstrated across tumour types comprising our core validation set. Total number of samples analysed for each tumour type is indicated in brackets. Only tumour types with 5 or more samples are shown.

ASPS: alveolar soft part sarcoma; CB: chondroblastoma; CHORD: chordoma; CSA: chondrosarcoma; CSA-CC: clear cell chondrosarcoma; CSA-MES: mesenchymal chondrosarcoma; Ctrl: control; ES: epithelioid sarcoma; FDY: fibrous dysplasia; GCTB: Giant cell of Tumour Bone; LMS: leiomyosarcoma; MFS: myxofibrosarcoma; MPNST: malignant peripheral nerve sheath tumours; NFB: neurofibroma OB: osteoblastoma; OS (HG): high-grade osteosarcoma; Pleo LPS: pleomorphic leiomyosarcoma; RMS: Rhabdomyosarcoma; SYSA: synovial sarcoma; USARC: undifferentiated sarcoma


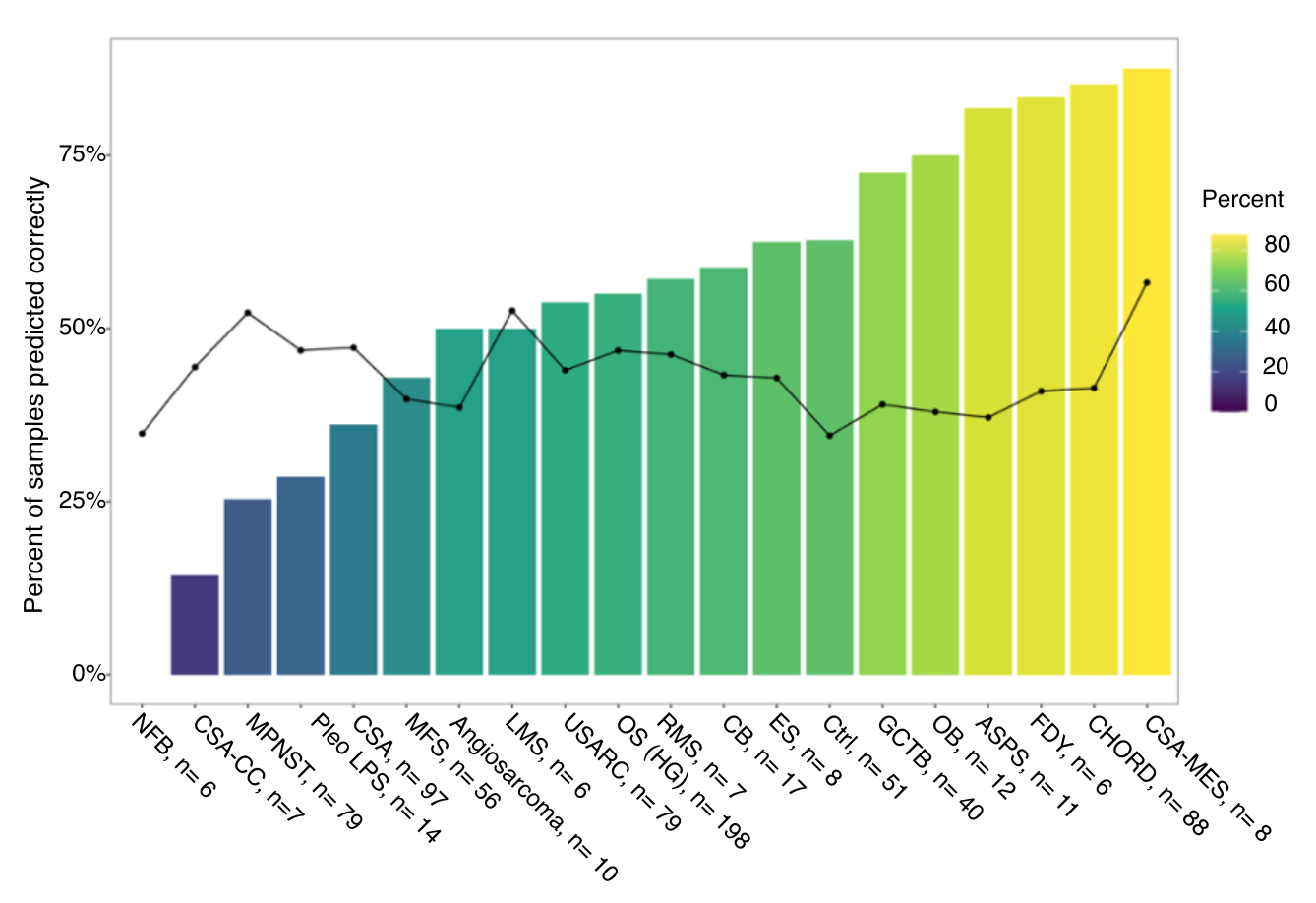


79

**Figure S6. Proportion of cases per subtype predicted to the correct methylation class is independent of tumour purity.** Only subtypes within the core validation cohort with more than five samples were included in this analysis. Bars indicate proportion of samples in each subtype predicted to the matching methylation class. The black line demonstrates median tumour purity (%) for each subtype.

ASPS: alveolar soft part sarcoma; CB: chondroblastoma; CHORD: chordoma; CSA: chondrosarcoma; CSA-CC: clear cell chondrosarcoma; CSA-MES: mesenchymal chondrosarcoma; Ctrl: control; ES: epithelioid sarcoma; FDY: fibrous dysplasia; GCTB: Giant cell of Tumour Bone; LMS: leiomyosarcoma; MFS: myxofibrosarcoma; MPNST: malignant peripheral nerve sheath tumours; NFB: neurofibroma OB: osteoblastoma; OS (HG): high-grade osteosarcoma; Pleo LPS: pleomorphic leiomyosarcoma; SYSA: synovial sarcoma; USARC: undifferentiated sarcoma


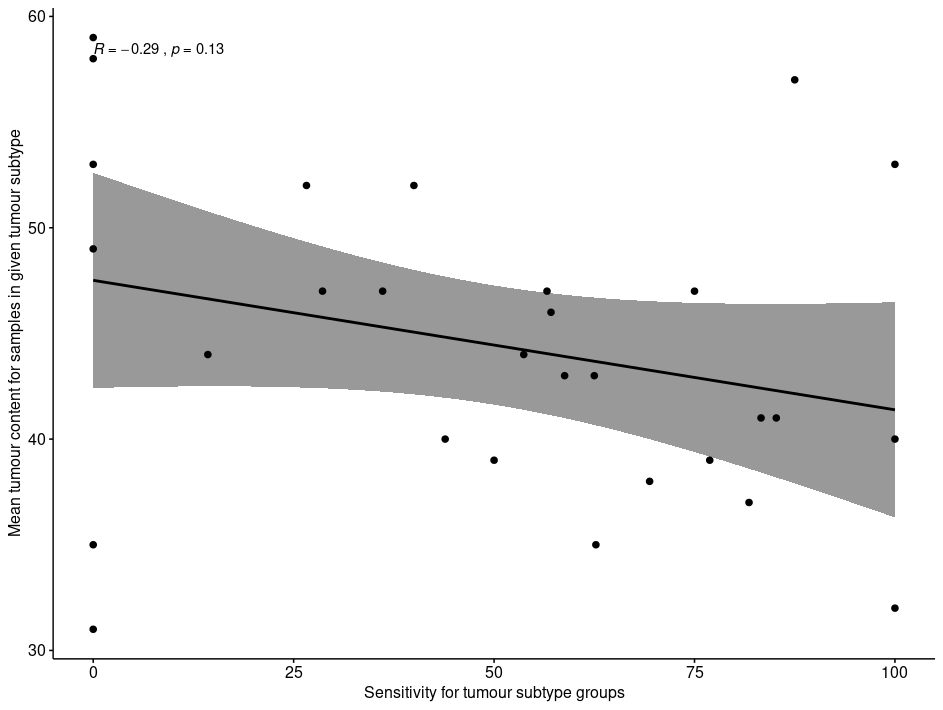


**Figure S7. Correlation plot showing no clear correlation between estimated tumour purity and the proportion of cases predicted correctly (sensitivity) per subtype.**

**Figure S8. Non-random distribution of the methylation chip type (450K vs EPIC arrays) associated with the different tumour types.**

AFH: Angiomatoid fibrous histiocytoma, ASPS: Alveolar soft part sarcoma, BCOR: BCOR-rearranged sarcoma, CB: chondroblastoma, CHORD: chordoma, CIC: CIC-rearranged sarcoma, CSA: chondrosarcoma; Ctrl: controls (blood or normal tissue), DFSP: Dermatofibrosarcoma protuberans, ES: epithelioid sarcoma, FDY: fibrous dysplasia, GCTB: Giant cell of Tumour Bone, IFS: Infantile fibrosarcoma, LMS: leiomyosarcoma, MEL (CUT): cutaneous melanoma, MFS: myxofibrosarcoma, MPNST: malignant peripheral nerve sheath tumours, NFB: neurofibroma, OB: osteoblastoma, OFMT:

Ossifying fibromyxoid tumour, OS (HG): high-grade osteosarcoma, Pleo LPS: pleomorphic leiomyosarcoma, RMS: Rhabdomyosarcoma, Sarcoma NOS: sarcoma not otherwise specified, SBRCT: small-blue round cell sarcoma, SEF: Sclerosing epithelioid fibrosarcoma, SFT: Solitary fibrous tumour, SYSA: synovial sarcoma, USARC: undifferentiated sarcoma, WDLS/DDLS: Liposarcoma.

**
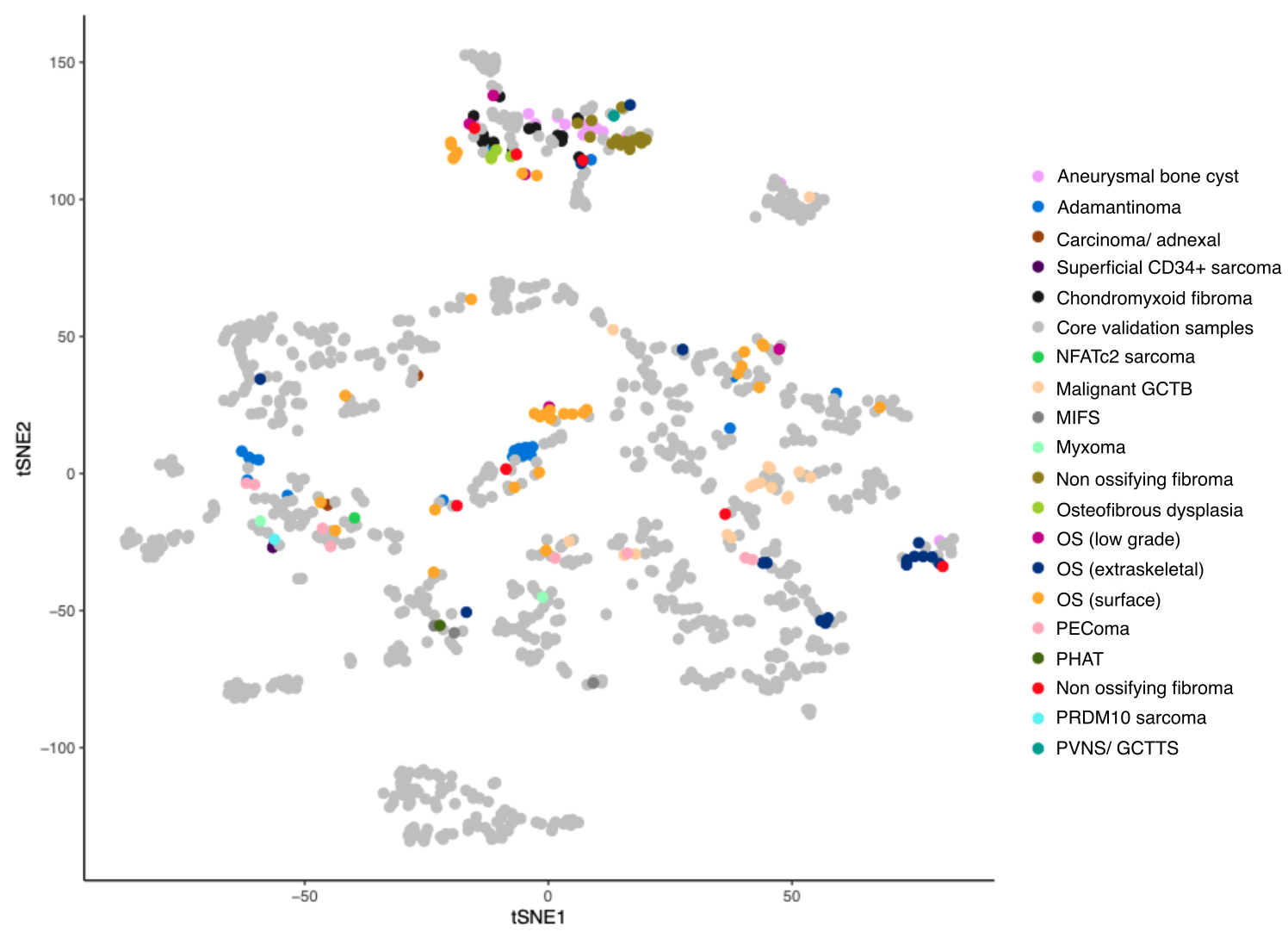
**

**Figure S9. t-distributed stochastic neighbour embedding (t-SNE) showing the clustering of the unrepresented samples.**

NFATc2 sarcoma: EWSR1-NFATC2 rearranged sarcoma, GCTB: giant cell tumour of bone, MIFS: myxoinflammatory fibroblastic sarcoma, OS: osteosarcoma, PEComa: perivascular epithelioid cell tumour, PHAT: pleomorphic hyalinising angiectatic tumour, PMT: phosphaturic mesenchymal tumour, PRDM10 sarcoma: PRDM10-rearranged soft tissue tumour, tenosynovial giant cell tumour.
